# Evaluation of Automated Multiclass Fluid Segmentation in Optical Coherence Tomography Images Using the Pegasus Fluid Segmentation Algorithms

**DOI:** 10.1167/tvst.10.1.27

**Published:** 2021-01-15

**Authors:** Louise Terry, Sameer Trikha, Kanwal K. Bhatia, Mark S. Graham, Ashley Wood

**Affiliations:** 1 School of Optometry and Vision Sciences, Cardiff University, Cardiff, UK; 2 King's College Hospital NHS Foundation Trust, London, UK; 3 Metalynx Ltd, London, UK; 4 School of Biomedical Engineering & Imaging Sciences, King's College London, London, UK

**Keywords:** optical coherence tomography, fluid segmentation, age-related macular degeneration, diabetic macular edema, clinical decision support

## Abstract

**Purpose:**

To evaluate the performance of the Pegasus-OCT (Visulytix Ltd) multiclass automated fluid segmentation algorithms on independent spectral domain optical coherence tomography data sets.

**Methods:**

The Pegasus automated fluid segmentation algorithms were applied to three data sets with edematous pathology, comprising 750, 600, and 110 b-scans, respectively. Intraretinal fluid (IRF), sub-retinal fluid (SRF), and pigment epithelial detachment (PED) were automatically segmented by Pegasus-OCT for each b-scan where ground truth from data set owners was available. Detection performance was assessed by calculating sensitivities and specificities, while Dice coefficients were used to assess agreement between the segmentation methods.

**Results:**

For two data sets, IRF detection yielded promising sensitivities (0.98 and 0.94, respectively) and specificities (1.00 and 0.98) but less consistent agreement with the ground truth (dice coefficients 0.81 and 0.59); likewise, SRF detection showed high sensitivity (0.86 and 0.98) and specificity (0.83 and 0.89) but less consistent agreement (0.59 and 0.78). PED detection on the first data set showed moderate agreement (0.66) with high sensitivity (0.97) and specificity (0.98). IRF detection in a third data set yielded less favorable agreement (0.46–0.57) and sensitivity (0.59–0.68), attributed to image quality and ground truth grader discordance.

**Conclusions:**

The Pegasus automated fluid segmentation algorithms were able to detect IRF, SRF, and PED in SD-OCT b-scans acquired across multiple independent data sets. Dice coefficients and sensitivity and specificity values indicate the potential for application to automated detection and monitoring of retinal diseases such as age-related macular degeneration and diabetic macular edema.

**Translational Relevance:**

The potential of Pegasus-OCT for automated fluid quantification and differentiation of IRF, SRF, and PED in OCT images has application to both clinical practice and research.

## Introduction

Optical coherence tomography (OCT) has become an essential diagnostic and management tool in ophthalmic eye care, providing high-resolution three-dimensional imaging of the neural retina, retinal pigment epithelium, and adjacent anatomic structures. The visualization of fluid within these tissues is a pivotal property of the technology and clinically highly important. The detection and quantification of fluid (edema), including the presence, size, location, and subsequent changes, is a key consideration in treatment management^1^^–^^3^ and clinical protocols^4^^–^^6^ for numerous ocular conditions, including age-related macular degeneration (AMD), diabetic macular edema (DME), and central serous retinopathy (CSR).

Given the importance of fluid evaluation for disease detection, as well as monitoring and assessing responses to interventions, ophthalmologists are keenly interested in accurate automated segmentation and differentiation between fluid types. It is unsurprising that extensive efforts have been made to develop and improve automated systems for the detection, quantification, and differentiation of fluid types from OCT imagery.^7^^–^^11^

Pegasus-OCT v1.0 (Visulytix Ltd, London, UK) is a clinical decision support system for detecting disease from macular OCT scans intended for use with images from different OCT devices. Pegasus primarily uses deep learning technologies to identify images with anomalous features that may be indicative of disease to enable classification into disease groups.^12^

Additionally, Pegasus-OCT incorporates automated multiclass fluid segmentation algorithms designed to detect and segment three clinical subtypes of fluid: intraretinal fluid (IRF), subretinal fluid (SRF), and pigment epithelial detachment (PED). IRF is characterized as a hyporeflective space (cystoid fluid) located within the neural retinal tissue, SRF as a hyporeflective space located between the hyperreflective retinal pigment epithelium (RPE) and the overlying neural retina, and PED as a hyporeflective space located between the RPE and underlying Bruch's membrane, visible on the OCT as the anterior of the choroidal vascular layer. Despite extensive investigation of fluid segmentation of OCT images, literature reporting systems capable of categorizing fluid by type on independent, unseen data sets is limited to date and often does not differentiate PED as a distinct subtype.^7^^,^^8^

This article provides an evaluation of the automated fluid detection capabilities of Pegasus-OCT. Three independent publicly available OCT data sets are used to assess the accuracy of the Pegasus fluid segmentation algorithms against manual expert-evaluated segmentation, as well as the sensitivity and specificity of the software in patients for the detection of IRF, SRF, and PED.

## Methods

### Pegasus Automated Fluid Segmentation Algorithms

Pegasus-OCT has automated fluid segmentation algorithms, aimed at delineating fluid within and beneath the retinal structures. This uses three convolutional neural network (CNN) models: one to predict SRF, one IRF, and one PED. The models were trained using the DeeplabV3+ architecture,^13^ allowing the system to identify different instances of the same class in the same image. To minimize the effect of high-frequency image noise (speckle), areas of detected fluid were designated to be a minimum of 5 pixels in size. Training was conducted using large heterogeneous real-world data sets from multiple clinical organizations across the world and incorporated both normal and abnormal scans. The abnormal OCT scans included retinal pathologies such as AMD, DME, macular hole, and epiretinal membrane. Training was conducted using images that were graded by between one and five UK board-certified ophthalmologists providing the ground truth. These images originated from standard spectral domain OCT devices, including the Heidelberg Spectralis (Heidelberg, Germany), Zeiss Cirrus (Dublin, California, USA), Nidek (Gamagori, Japan), and Topcon Maestro (Tokyo, Japan).

### Image Data Sets

Three publicly available data sets^7^^,^^14^^–^^16^ of Spectralis OCT images were used to evaluate the fluid segmentation algorithms of the Pegasus system. Each of the data sets selected included manual labeling of fluid features within individual OCT b-scans by the data set owners and was not used in the development of the Pegasus fluid segmentation algorithms, providing a ground truth for testing the performance of the algorithms. The data sets were used “as is” to avoid introducing any selection bias that may occur if images were removed based on “image quality” or other criteria. The characteristics of the data sets included are described below and summarized in Table 1; the most significant differences between data sets are fluid types provided in the ground truth and number of b-scans per participant.

**Table 1.** Characteristics of the Independent Evaluation Data Sets Used in This Study

Data Set	Source	OCT	Country of Acquisition	Number of B-Scans (Subjects)	Disease State	Ground Truth Labeling	Fluid Subtype Evaluated
A	Images from Kermany et al. (2018)^16^ Labeled by Lu et al. (2019)^7^	Heidelberg Spectralis	USA, China	750 (530)	DME Drusen Normal	3 tiers of trained raters, subset validated by 2 senior retinal specialists	IRF SRF PED
B	Rashno et al. (2017)^15^	Heidelberg Spectralis	USA	600 (24)	Exudative AMD	2 ophthalmologists	IRF SRF
C	Chiu et al. (2015)^14^	Heidelberg Spectralis	USA	110 (10)	DME	2 ophthalmologists; DOCTRAP v50.9	IRF

Data set A^7^^,^^16^ was obtained from 530 participants with AMD, DME, or healthy controls. Between 1 and 13 individual b-scans (see Lu et al.^7^) were extracted from each volume to produce a data set containing 750 individual b-scans (250 AMD, 250 DME, and 250 normal). For each b-scan, regions of IRF, SRF, or PED were manually segmented, if present, by three trained raters and subsequently reviewed by two ophthalmology clinical scientists providing a ground truth for each OCT b-scan.

Data set B was obtained from 24 participants with exudative AMD, comprising 25 OCT b-scans per participant.^15^ Two ophthalmologists identified and segmented regions of IRF and SRF manually, which served as the ground truth. No segmentation data for PED were available for this data set.

Data set C was obtained from 11 participants with severe DME, comprising 10 OCT b-scans per participant.^14^ Two ophthalmologists independently identified and segmented regions of IRF manually, which served as the ground truth. No segmentation data for SRF or PED were available for this data set. Due to the availability of manual segmentations from each grader, it was possible to also evaluate intergrader discrepancies in fluid segmentation for this data set.

### Analysis

The analysis was performed on masks generated for every OCT b-scan within each data set representing the location of fluid identified by the Pegasus system. These were then compared to a further set of masks containing the “ground truth” fluid locations within every b-scan as determined by the data set owners. All analyses were performed using custom scripts in MATLAB R2019a (The MathWorks, Inc., Natick, MA, USA).

Dice coefficients^17^ were used to assess agreement between the manual segmentation provided by the data set owners and the Pegasus system. These were calculated as follows:

$$\begin{array}{c} \mathrm{Dice}\;\text{coefficient}=2\left(A\cap B\right)/\left(A+B\right), \end{array}$$

where A represents the manual segmentation, B represents the automated segmentation, and A∩B is the number of common pixels between the two sets.

Sensitivity and specificity values were calculated based on comparing the presence or absence of fluid against the ground truth for each individual b-scan within the overall data set or for individual eyes where sufficient data were available. Sensitivity was determined based on the number of slices where any fluid was identified by Pegasus that corresponded to a slice where the ground truth also identified any fluid (true positive; TP), divided by this number (TP), plus the number of slices where Pegasus did not identify any fluid contrary to the ground truth (false negative; FN), that is, sensitivity = TP/(TP + FN).

Specificity was determined based on the number of slices where neither Pegasus nor the ground truth identified any fluid (true negative; TN), divided by this number (TN), plus the number of slices where Pegasus identified fluid contrary to the ground truth (false positive; FP), that is, specificity = TN/(TN + FP).

Dice coefficients, sensitivity, and specificity were determined separately for each data set overall (A, B, and C) and “per eye” for data sets B and C, where volumes contained sufficient b-scans (*n* = 11 and *n* = 24, respectively) to perform this within-eye analysis. Fluid type (e.g., SFR, IRF, and PED) was analyzed separately for each data set where ground truth data (manual segmentation) was available. For data set B, the OCT volume for every eye contained b-scans with at least one subtype of fluid in the ground truth, but not all volumes contained both fluid subtypes (i.e., IRF and SRF); hence, it was not possible to calculate Dice coefficients, sensitivity, or specificity for these individual eyes (see Supplementary Table S1). IRF was evaluated on all three data sets, SRF was evaluated on data sets A and B, and PED was evaluated on data set A only.

## Results

**Figure 1.**
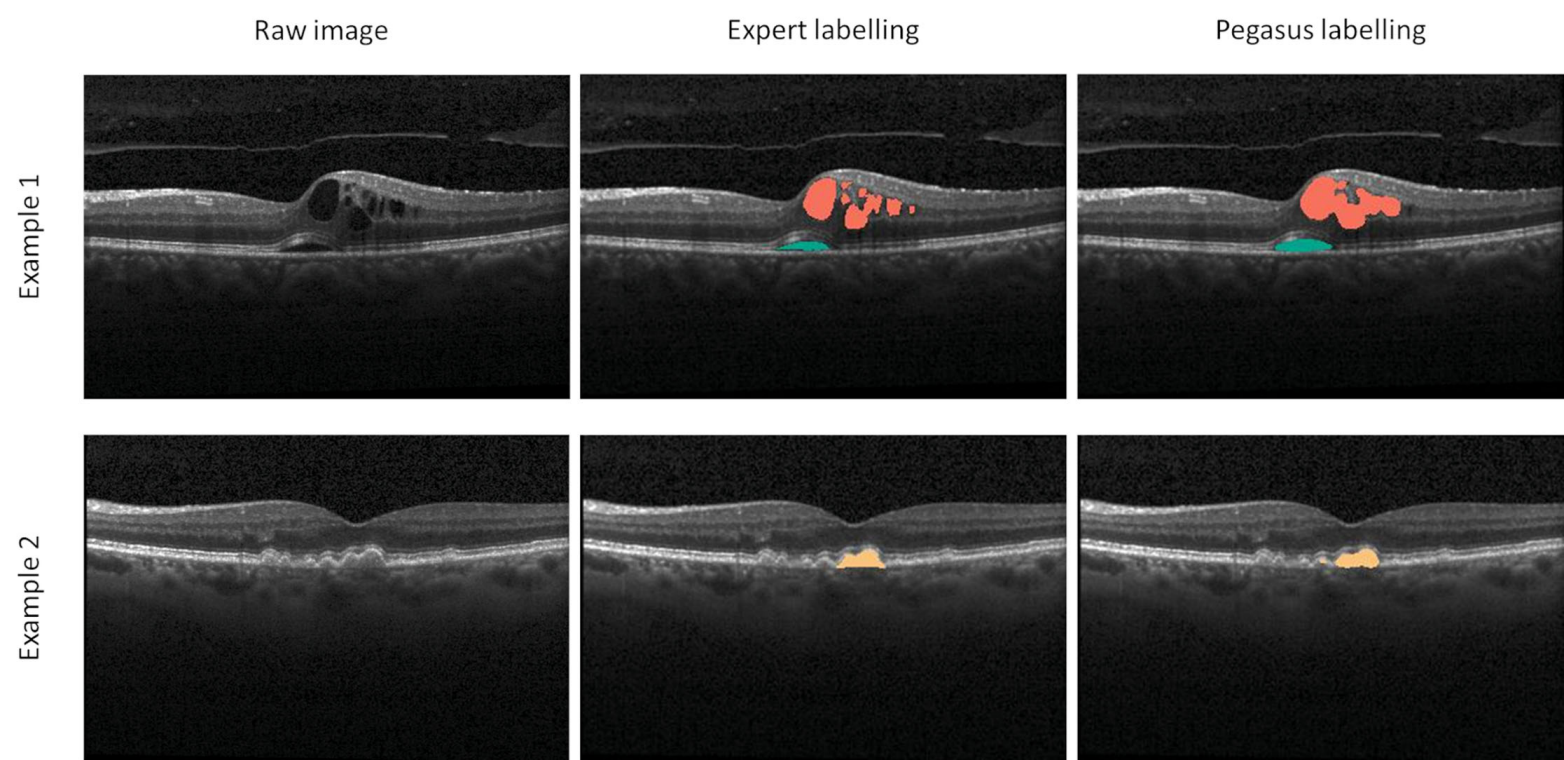
Example comparison between the expert and automated fluid segmentation (examples taken from data set A). Intraretinal fluid is shown in *red*; subretinal fluid in *green*; pigment epithelial detachment in *orange*.

All three data sets underwent automated fluid segmentation using the Pegasus system (total *n* = 1460 b-scans from three independent data sets). Examples of successful segmentation of IRF, SFR, and PED can be seen in Figure 1. The Dice coefficients, sensitivity, and specificity separated by fluid type (IRF, SRF, and PED) and data set (A, B, and C) are shown in Table 2. The performance of the Pegasus system for the individual eyes in data sets B and C can be found in Supplementary Tables S1 and S2, respectively.

**Table 2.** Summary of Dice Coefficients, Sensitivity, and Specificity for the Three Fluid Features, Across All Data Sets

Data Set	Dice Coefficient, Mean	Dice Coefficient, Median (IQR)	Sensitivity	Specificity
Intraretinal fluid				
A	0.78	0.81 (0.12)	0.98	1.00
B	0.54	0.59 (0.29)	0.94	0.89
C;(Expert 1 vs Pegasus)	0.57	0.70 (0.84)	0.68	1.00
C;(Expert 2 vs Pegasus)	0.46	0.51 (0.79)	0.59	0.92
C;(Expert 1 vs 2)	0.59	0.68 (0.34)	0.94	0.59
Subretinal fluid				
A	0.59	0.70 (0.31)	0.86	0.83
B	0.78	0.84 (0.11)	0.98	0.89
Pigment epithelial detachment				
A	0.66	0.71 (0.23)	0.97	0.98

### Intraretinal Fluid

The Pegasus automated fluid segmentation algorithms showed excellent sensitivity (0.98 and 0.94) and specificity (1.00 and 0.89) for data sets A and B, respectively, but a relatively low sensitivity for data set C (∼0.64). Within data set C, performance was particularly poor on subject 9, in whom there was very little overlap between the experts and automated segmentation. However, on a per-eye level for this data set (all 11 b-scans per eye considered together), all eyes were correctly identified as having IRF on at least one b-scan.

**Figure 2.**
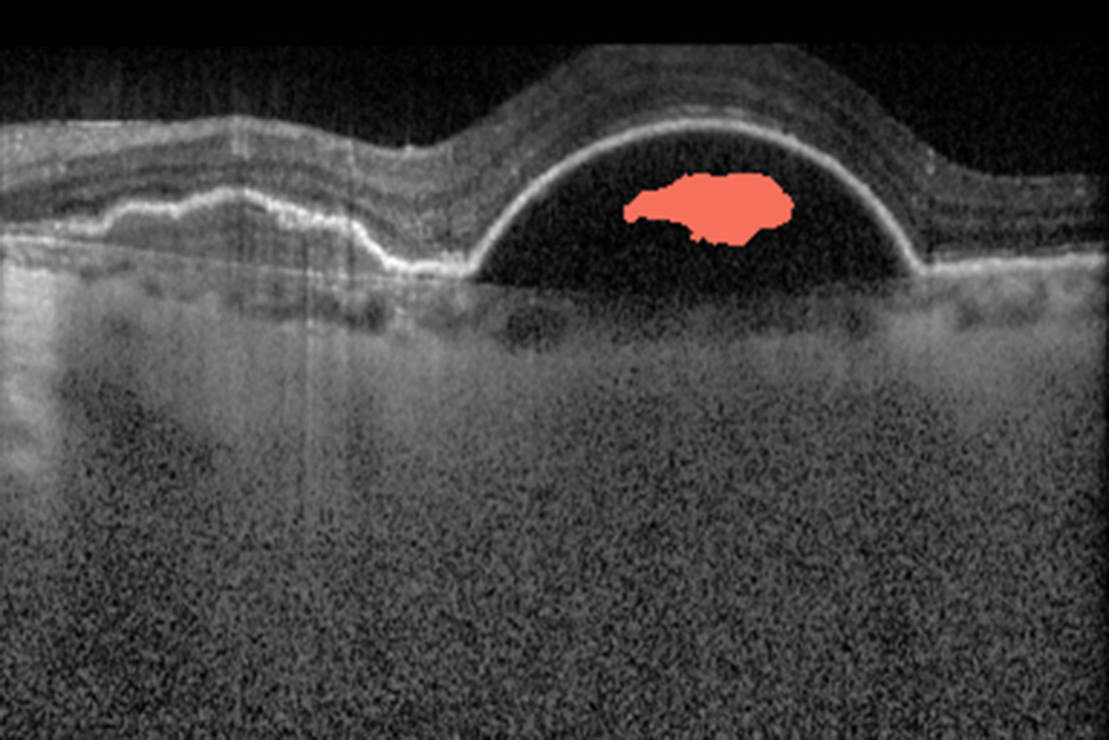
In data set B, the automated segmentation incorrectly labeled areas within a serous pigment epithelial detachment as intraretinal fluid.

For data set B, only 7 of the 24 eyes contained IRF in the expert grading, but the Pegasus system identified 15 of the eyes as positive for IRF, yielding a high false-positive rate. Several of these were identified on a single b-scan only per eye, and 3 were cases of serous PED incorrectly labeled by the algorithms as IRF (Fig. 2). There were no eyes in which the algorithms failed to detect IRF where it had been identified by the two experts (i.e., false negative rate = 0). On a per-eye level for data sets B and C, IRF detection had perfect sensitivity (1 for both), but the specificity for data set B was poorer (0.53). It was not possible to assess specificity for data set C on a per-eye level, since there were no true negatives for IRF.

A comparison of the manual segmentations performed by the two expert graders in data set C showed limited agreement (mean Dice coefficient 0.59; comparable to that of the Pegasus software, at 0.57 and 0.45 for experts 1 and 2, respectively). Sensitivity for fluid detection was high between experts (0.94), but specificity was low (0.59).

### Subretinal Fluid

**Figure 3.**
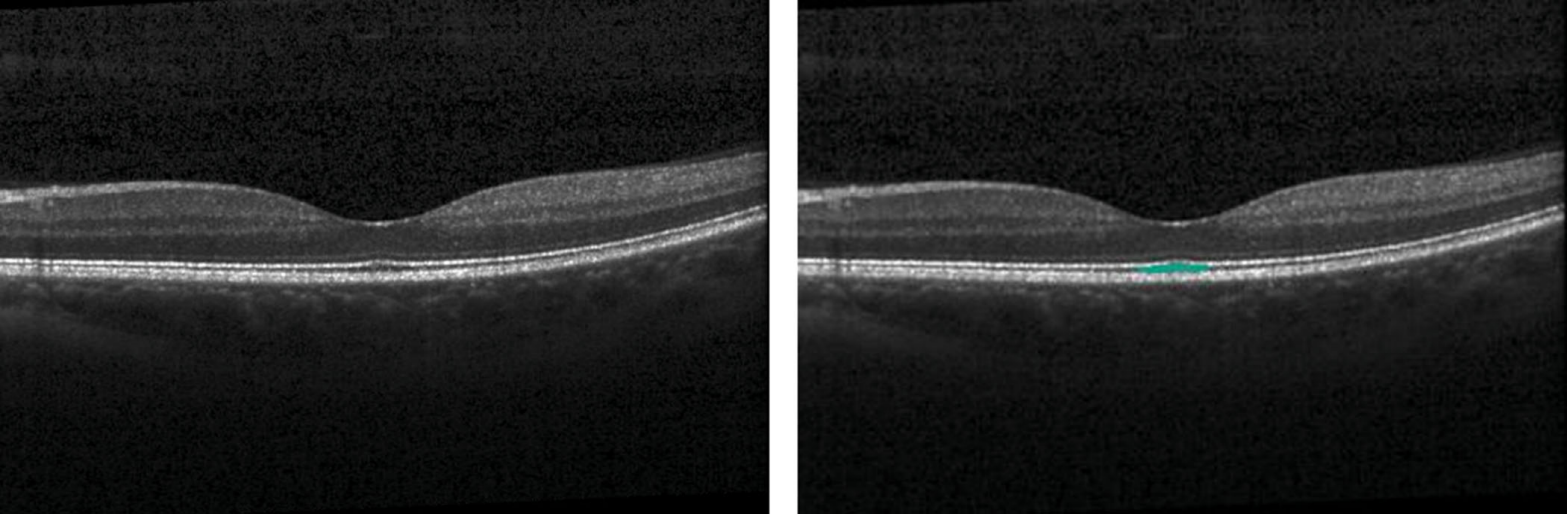
For data set A, the physiologically thickened photoreceptor outer segment layer beneath the foveal pit (*left*—original image) was mislabeled by the automated algorithm subretinal fluid (*right*—labeled image), limiting the specificity.

The Pegasus fluid segmentation algorithms achieved better performance for data set B than A for SRF, achieving a high sensitivity (0.98), specificity (0.89), and mean Dice coefficient (0.78). For data set A, the algorithms labeled the physiologically thickened layer of outer segments beneath the foveal pit as SRF on several of the eyes without pathology (Fig. 3), limiting the specificity in this analysis (0.83). On a per-eye level for data set B, 20 of the 24 eyes contained SRF in the expert grading. The Pegasus system identified 22 of the eyes as positive for SRF, but both false-positive cases were in images containing PED. There were no eyes in which the algorithms failed to detect SRF where it had been identified by the expert grader (sensitivity = 1). The specificity for this data set was low (0.5), although there were only 4 eyes without SRF in the data set, 3 of which were cases of PED.

### Pigment Epithelial Detachment

Evaluation of PED was only possible on data set A, since it was the only data set labeled for this feature by the expert graders. The sensitivity and specificity were excellent (0.97 and 0.98, respectively), with reasonable agreement in segmentation between the expert and automated segmentations (mean Dice coefficient 0.66). Despite being the largest data set available, a per-eye level analysis was not possible, as there were too few b-scans available per eye (only a single b-scan in many cases).

## Discussion

In this article, we assessed the detection and segmentation performance of the Pegasus automated fluid segmentation algorithms on three existing independent OCT data sets. These data sets contained up to three distinct types of fluid: IRF, SRF, and PED. Pegasus performance on each data set was benchmarked against expert-derived ground truth, with the algorithms providing good but not always consistent performance across the three data sets and fluid subtypes.

Overall, Pegasus's ability for automated detection of all three fluid types based on sensitivity and specificity was favorable and compares well to figures reported in contemporary literature. For data set A, performance was very high for IRF and PED while slightly poorer for SRF, the same pattern presented by Lu et al.^7^ Our results outperform those of Rashno et al.^15^ for data set B in terms of sensitivity (0.81 versus 0.96) with a similar specificity found (0.91 versus 0.89). No sensitivity or specificity values to allow comparison were presented by Chiu et al.^14^ for data set C.

Pegasus provided promising fluid segmentation performance, based on Dice coefficients, across the three fluid types evaluated. Data set A uniquely allowed evaluation on all three fluid subtypes, but the mean Dice coefficients were lower in the present study than those presented by Lu and colleagues^7^ (0.59–0.78 versus 0.75–0.9). Both studies reported the best performance for IRF compared to the other two fluid subtypes, and likewise for data set B, although only a single Dice coefficient of 0.82 was reported by Rashno et al.,^15^ despite the investigation of both IRF and SRF. For data set C, our Dice coefficients were comparable to those reported in the literature,^14^ including a limited interobserver performance during ground truth labeling. The authors attribute this to reduced image quality and the severity of DME included.^14^

The RETOUCH project presents the performance of eight different deep learning algorithms for (a) automated fluid detection and (b) automated fluid segmentation.^18^ A comprehensive review of algorithm performance in these tasks for each of the three fluid subtypes, plus a table of other relevant works, can be found elsewhere. For the fluid detection task, the results were comparable for PED and SRF (on data set B), although Pegasus relatively outperformed on IRF (on data sets A and B) and underperformed on SRF (on data set A) and IRF (on data set C). For the fluid segmentation task, the mean Dice coefficients for Spectralis images were 0.69 for IRF, 0.57 for SRF, and 0.68 for PED. Again, these are comparable to Pegasus's average performance across the three data sets. For this task, Pegasus relatively outperformed on SRF (particularly on data set B) and IRF (on data set A) but underperformed on IRF (on data sets B and C). Similarly to RETOUCH, the Pegasus system yielded a high performance for detection of PED, likely due to its distinct appearance and location in relation to the RPE.

It should be noted that all OCT images used in this evaluation were acquired with a Spectralis SD-OCT (Heidelberg Engineering). We are therefore unable to comment on the performance of these fluid segmentation algorithms on images acquired with other OCT devices. However, the Pegasus-OCT system is intended to be OCT device independent and has been developed and trained based on multiplatform OCT data. Of note, RETOUCH^18^ found that best performances were achieved when neural networks were trained on data from each OCT device separately, highlighting the trade-off between device-specific performance and algorithm generalizability. The results of the present study are comparable to much of the contemporary published literature, including algorithms trained on data from single OCT platforms, which demonstrates the promise of the Pegasus system.

The interexpert comparison for data set C provides a mean Dice coefficient (0.59) that was not appreciably higher than either expert grading versus the Pegasus segmentation (0.57 and 0.45 for experts 1 and 2, respectively). It should be noted that the data set owners attributed their expert graders’ performance to “lower image quality and severe DME pathology present.”^14^ The interexpert agreement for this data set is notably lower than other contemporary studies with Dice coefficients reported in the region of ∼0.75,^9^^,^^18^ although not uniquely so,^19^ with the severity of retinal pathology as an important factor. This suggests the interexpert variation and its influence on the ground truth is likely to be a pertinent factor in the Pegasus system's performance. The greater differences between mean and median Dice coefficients (see Table 2), and wider Inter Quartile Range (IQR) attributable to this data set (C), compared to A and B, would be consistent with increased outliers due to poor agreement on individual scans. Furthermore, the higher sensitivity and limited specificity values for the interexpert comparison in relation to the Pegasus system could speculatively be explained if an internal “bias” within the expert clinicians for overdetecting (rather than underdetecting) retinal fluid was present. The clinical experience of each expert and how they balance the relative risk of a false-positive versus a false-negative result on clinical outcomes may influence their approach to the manual fluid segmentation of the images.

Visual inspection of labeled images suggests expert graders provide a more “granular” demarcation of the regions of fluid compared to the algorithm that appears smoother (Fig. 1); these discrepancies, while small, would have contributed to reducing the Dice indices (agreement). The absolute fluid area is also likely to be affected; therefore, consistency of method (i.e., not used interchangeably) would be advised in applications such as progression monitoring, where small absolute differences may have clinical significance.

We also caution that the data sets used for validation in this study consisted of eyes with the most common pathologies characterized by fluid (AMD and DME). Fluid segmentation performance in eyes with other retinal pathologies or comorbidities (e.g., macular hole, epiretinal membrane, central serous retinopathy) was not evaluated due to restricted data availability for these conditions, which were independent of the algorithm's training.

A “true” sensitivity and specificity was only possible to evaluate on data set A, since this data set was the only of the three to contain b-scans from eyes entirely absent of retinal disease. Data sets B and C did not contain any healthy eyes, confirmed as having no fluid visible on any b-scan by expert graders. For these two data sets, performance metrics were evaluated on a “per-b-scan” basis only, rather than a “per-eye” basis as in data set A, which would be the usual clinical scenario.

The number of b-scans per eye included in the analysis also differed between data sets. This should be considered when interpreting these results, since data sets with a higher ratio of number of b-scans to number of eyes (e.g., data set B) will likely have greater homogeneity, which may influence algorithm performance. Data set A had by far the largest number of eyes and included both AMD and DME. It might therefore be considered that this data set provided the greatest challenge for the Pegasus-OCT system in variation of disease appearance.

This study is not typical of a classical automated feature detection task (i.e., presence or absence of a distinct image feature); rather, it represents a more complex feature detection and subtype classification task, with the subtypes having a similar appearance in many cases. This may be particularly problematic in low-quality images, where dark or low-contrast portions of the image resulting from signal loss (e.g., from media opacities) may be mistakenly identified as fluid. This, in part, may explain the limited performance of the algorithm for certain metrics and fluid subtypes.

Overall, the Pegasus-OCT fluid segmentation algorithms were shown to be capable of detecting IRF, SRF, and PED within spectral domain OCT images of independent data sets from multiple sites. The competitive performance against other published methods demonstrates the potential of this approach as a clinical tool for the autonomous detection and monitoring of retinal disease such as AMD and DME.

## Supplementary Material

Supplement 1
