# Supplementary material for: Evaluation of Automated Multiclass Fluid Segmentation in Optical Coherence Tomography Images Using the Pegasus Fluid Segmentation Algorithms
Source: Transl Vis Sci Technol. 2021 Jan 15;10(1):27. doi: 10.1167/tvst.10.1.27 (PMC9354552; doi:10.1167/tvst.10.1.27)
Supplement: Supplement 1 [file tvst-10-1-27_s001.pdf]

Supplementary Table 1. Average Dice coefficients, sensitivity, and specificity for intra-retinal fluid (IRF) and sub-retinal fluid (SRF) segmentation for each eye in Dataset B.

	Intra-retinal Fluid			Sub-retinal Fluid		
**Subject**	Dice Coefficient	Sensitivity	Specificity	Dice Coefficient	Sensitivity	Specificity
1	-	-	-	0.80	1.00	0.83
2	-	-	-	0.81	1.00	0.93
3	-	-	-	0.80	1.00	0.70
4	0.59	1.00	0.86	0.86	1.00	0.83
5	0.36	0.80	0.85	0.60	0.94	0.56
6	-	-	-	0.74	1.00	1.00
7	0.34	0.80	0.90	-	-	-
8	-	-	-	0.80	1.00	1.00
9	-	-	-	0.78	1.00	1.00
10	0.62	1.00	1.00	0.77	1.00	0.90
11	-	-	-	0.82	1.00	0.86
12	-	-	-	0.85	1.00	0.80
13	-	-	-	0.83	1.00	1.00
14	-	-	-	0.79	1.00	0.88
15	0.51	1.00	0.75	0.75	1.00	0.83
16	-	-	-	0.80	1.00	1.00
17	-	-	-	0.85	1.00	1.00
18	-	-	-	0.86	1.00	0.69
19	-	-	-	0.76	0.93	1.00
20	0.57	1.00	1.00	0.58	0.75	1.00
21	-	-	-	-	-	-
22	-	-	-	-	-	-
23	-	-	-	-	-	-
24	0.79	1.00	0.88	0.83	1.00	1.00

Null values were returned where either the ‘ground truth’ or Pegasus did not detect any fluid, preventing the calculation of a Dice coefficient, sensitivity and specificity.

Supplementary Table 2. Average Dice coefficients and sensitivity for intra-retinal fluid (IRF) segmentation for each eye* in Dataset C. Note, specificity was not calculated as the dataset does not contain 'true negative' data.

	**Dice Coefficient**			**Sensitivity**		
**Subject**	Expert 1	Expert 2	Average	Expert 1	Expert 2	Average
1	0.68	0.48	**0.58**	0.78	-	**0.78**
2	0.60	0.60	**0.60**	0.57	0.57	**0.57**
3	0.72	0.43	**0.58**	1.00	0.63	**0.81**
4	0.50	0.55	**0.53**	0.56	0.63	**0.59**
5	0.48	0.47	**0.48**	0.60	0.60	**0.60**
6	0.50	0.50	**0.50**	0.70	0.70	**0.70**
7	0.83	0.75	**0.79**	0.75	0.67	**0.71**
8	0.56	0.43	**0.49**	0.75	0.63	**0.69**
9	0.39	0.01	**0.20**	0.14	-	**0.14**
10	0.45	0.34	**0.40**	1.00	0.90	**0.95**

*Null values were returned where either the ground truth or Pegasus did not detect any fluid, preventing the calculation of a Dice coefficient, sensitivity and specificity.
